# Antifungal activity of berberine hydrochloride and palmatine hydrochloride against *Microsporum canis* -induced dermatitis in rabbits and underlying mechanism

**DOI:** 10.1186/s12906-015-0680-x

**Published:** 2015-06-09

**Authors:** Chen-Wen Xiao, Quan-An Ji, Qiang Wei, Yan Liu, Guo-Lian Bao

**Affiliations:** Institute of Animal Husbandry and Veterinary Science, Zhejiang Academy of Agricultural Sciences, ShiQiao Road 145, Hangzhou, Zhejiang 310021 People’s Republic of China

**Keywords:** *Phellodendron amurense*, berberine hydrochloride, Palmatine hydrochloride, Antifungal mechanism, *Microsporum canis*

## Abstract

**Background:**

*Phellodendron amurense*, exhibits antifungal activity mainly by bioactive components including berberine hydrochloride and palmatine hydrochloride. This study was conducted to evaluate the antifungal effects of berberine hydrochloride, palmatine hydrochloride, and a mixture of both substances against *Microsporum canis in vivo* and *in vitro*.

**Methods:**

The minimal inhibitory concentrations (MICs) of monomers and clotrimazole were determined using 1.5 % tryptic soy agar. The effects of these drugs on *Microsporum canis* growth was detected by determining dry weight. Transmission electron microscopy (TEM) was performed to observe the effect of chemicals on cell ultrastructure. Differential mRNA expressions of eight genes of *M. canis* treated with berberine or palmatine or their combination at different time points were determined by real-time PCR. NADH enzyme concentration was also detected. Clinical evaluation via *in-vivo* antifungal assay was also performed. Skin histology PAS staining was also carried out.

**Results:**

Results showed that MICs of berberine, palmatine and clotrimazole were 1, 1, and 0.015 mg/mL, respectively. No significant difference was observed among the growth curves of the three groups before 18 h was reached. TEM showed that these drugs could destroy the cell membrane and organelles of *M. canis* at different time points. After 30 h of incubation, relative mRNA expressions of the genes in the combined group were significantly higher than those in the other groups including the clotrimazole group (*P* < 0.05); Palmatine initially induced the mRNA up-regulation of PGAL4, FSH1, PQ-LRP, NADH1 and NDR in *M. canis*; by contrast, berberine maintained a high expression level of these genes to shorten fungal life cycle and eradicate *M. canis*. Clinical results showed that combined treatment was more effective than single administration of each monomer or clotrimazole. Hence, berberine mixed with palmatine significantly elicited antifungal activities and could be used to treat *M. canis* in rabbits.

**Conclusion:**

These results provide a comprehensive view of the mechanism of berberine and palmatine in anti-*M. canis* activity.

## Background

Dermatophytes are pathogenic fungi that can invade keratinized structures and infect skin, hair, and nails of humans and other animals [1]. In animal dermatomycosis, the most common pathogens are *Trichophyton mentagrophytes* [2], *Microsporum gypseum* and *Microsporum canis* [3, 4]. The species *M. canis* is found in humans and other animals; notably, *M. canis* is zoonotic in nature. *M. canis* is also known as one of the causes of dermatophytosis in rabbits [5, 6]. A total of 21 isolates of *M. canis* have been collected from rabbits with or without skin lesions [1].

Rabbit dermatomycosis is a kind of highly infectious zoonotic contact dermatitis. The disease mainly causes dandruff, hair removal, exudation, crusting, folliculitis, and itching [7]; This disease can also result in rabbit malnutrition, growth retardation, feed remuneration reduction and even death. Furthermore, dermatomycosis directly affects the quality of skin, reproductive performance, and survival rate of young rabbits. In many warrens, dermatomycosis occurs at an incidence rate of 30 % to 100 %, pup growth rate decreases by 20 % to 30 % and mortality rate ranges from 20 % to 40 % before weaning [8].

Dermatophytosis is treated by using various antifungal agents, such as clotrimazole, terbinafine, and ketoconazole [9]. However, drug resistance, toxicity, and drug-drug interactions limit the use of these treatments [10, 11]. Medicinal plants play an essential role in Chinese ethnoveterinary medicine [12] because these plants can effectively treat various ailments [13]. Approximately 40 % of the total medicinal consumption in China is attributed to traditional medicines [12]. Antimicrobial, fungicidal, and antioxidant properties of many therapeutic plant extracts have been widely reported [14]. These medicinal properties are caused by active chemical constituents in their roots, stems, leaves, seeds, and bark.

The bark of a *Phellodendron* tree has been used in traditional Chinese medicine for thousands of years. *P. amurense* is commonly used to treat gastroenteritis, abdominal pain or diarrhea, and various inflammatory diseases, including arthritis and dermatophytosis. The main bioactive components of *P. amurense* are berberine hydrochloride and palmatine hydrochloride [15]. Previous studies have implied a number of biological activities of berberine, including anti-secretory, anti-inflammatory, anti-bacterial, anti-malarial, anti-mycobacterial [16], anti-tumor and anti-cholesterol activities. Berberine and palmatine were found inhibited CYP1A1.1- and CYP1B1.1-catalyzed 7-ethoxyresorufin O-deethylation (EROD) activities. Kinetic analysis revealed that berberine noncompetitively inhibited EROD activities of CYP1A1.1 and CYP1B1.1, whereas palmatine and jatrorrhizine caused either competitive or mixed type of inhibition [17]. In previous study, berberine and palmatine were screened to determine their inhibitory activities various dermatophytes [18]; results revealed that berberine exhibited activity against *M. canis* (MICs, μg/mL >1000). To determine the antifungal mechanism of *Phellodendron amurense* against *M. canis*, we used berberine hydrochloride, palmatine hydrochloride and combined treatment *in vitro* and vivo experiments. Our results could provide a scientific basis for the treatment of skin diseases with natural drugs.

## Methods

### Berberine hydrochloride, palmatine hydrochloride and clotrimazole

Berberine hydrochloride (HPLC > 98 %, Lot Number: 20130306) and palmatine hydrochloride (HPLC > 98 %, Lot Number: 20130109) were purchased from Yuan Ye Biological Technology Co., Ltd, (Shanghai, China). Clotrimazole (99 % pure, Lot No. 23593-75-1) was purchased from BaDaTong Medical Company (TaiZhou, Zhejiang Province, China).

### *In vitro* antifungal effect of berberine hydrochloride and palmatine hydrochloride

#### Fungal organism

*M. canis* was isolated from dermopathic rabbits obtained from Shaoxing District. The presence of *M. canis* was confirmed by Institute of Internal Medicine at the Chinese Academy of Medical Sciences (Nanjing, China).

#### *In vitro* antifungal assay

Eumycetes were grown on tryptic soy agar plates at 28 °C for 4 d [19]. The cultured material was collected by scraping the agar surface with a sterilized loop, and then transferred to a glass tube containing normal saline solution. The suspension was vortexed for 60 s, and heavy particles were allowed to settle for 3 min to 5 min. The density of the suspension was adjusted spectrophotometrically to obtain a primary inoculum at a final concentration of 1.0 × 10^6^ CFU/mL in normal saline solution.

### Determination of the minimum inhibitory concentration (MIC)

MIC is defined as the lowest concentration of a compound required to visibly inhibit growth. To assess MIC, we used agar-diffusion method with slight modification [20]. In brief, serial amounts of berberine hydrochloride or palmatine hydrochloride (0, 50, 100, 150, 200, 250, and 300 mg) were dissolved in 10 mL of dimethyl sulphoxide and gently mixed with 100 mL of tryptic soy agar. Similar preparations were made using serial amounts of clotrimazole (i.e., 0, 0.5, 1.0, 1.5, 2.0, 2.5, and 3 mg) dissolved in 0.2 mL of dimethyl sulphoxide. Clotrimazole served as the positive control. These mixtures were then poured into sterile Petri dishes allowed to solidify and incubating at 45 °C for 15 min. The final concentrations of berberine or palmatine were 0, 0.5, 1.0, 1.5, 2.0, 2.5, and 3 mg/mL. The final concentrations of clotrimazole were 0, 0.005, 0.01, 0.015, 0.02, 0.025, and 0.03 mg/mL. Afterward, 1.0 × 10^6^ CFU/mL (0.1 mL) eumycete suspension was inoculated onto the Petri dishes and incubated with 60 % humidity at 28 °C for 72 h. In this study, MIC was defined as the lowest concentration of berberine hydrochloride, palmatine hydrochloride, or clotrimazole required to inhibit fungal growth. Each experiment was performed in duplicate.

### Growth curve using dry weight determination

The time- and concentration- dependent effects of berberine or palmatine on *M. canis* were determined using the method described by Alió *et al.* [21]. The eumycete culture (30,000 cells/mL) was diluted with 200 mL of tryptone soya broth and added to conical flasks containing known concentrations of berberine hydrochloride, palmatine hydrochloride, their combination (1 mg/mL or 1 mg + 1 mg/mL) or clotrimazole (0. 4 mg/mL). The cultures were then incubated at 37 °C with continuous shaking at 170 cycles/min for 72 h. Duplicate 1 mL aliquots of homogenized samples were removed from each conical flask and transferred to pre-weighed Eppendorf tubes. After centrifugation was performed 13,300 × *g* for 20 min, the sediments were dried in an oven at 60 °C. Differential weights were determined using an analytical balance. Data were recorded at 6, 18, 30, 42, and 54 h. Each experiment was performed in duplicate.

### Ultrastructural analysis by transmission electron microscopy (TEM)

TEM was performed to observe the effect of the extract on cellular ultrastructure, as previously described [22, 23] with slight modifications. Approximately 50 mL of *M. canis* cells (3 × 10^5^ CFU/mL) were treated with either berberine hydrochloride, palmatine hydrochloride, their combination (1 mg/mL or 1 mg + 1 mg/mL) or clotrimazole (0. 4 mg/mL) for 18 and 30 h. The treated cells were fixed in 2.5 % glutaraldehyde prepared in 0.1 M phosphate buffer solution at a constant pH of 7.0 for 4 h at room temperature and then washed trice with phosphate buffer solution. The treated cells were then post-fixed using 1 % osmium tetroxide for 1 h at room temperature. The specimens were washed trice, dehydrated using a graduated ethanol series (50 %, 70 %, 80 %, 90 %, 95 %, and 100 %) for 20 min, and embedded in Spurr’s resin. Thin sections of the specimens were cut using an ultramicrotome and stained first with uranyl acetate and then with lead citrate for 15 min. The sections were observed using a JEM-1230 TEM (JEOL Ltd., Japan).

### Inhibitory effect of berberine, palmatine, or combined treatment on the differential expression of M. canis-related genes

Approximately 1 mL of *M. canis* cells (5 × 10^9^ CFU /ml) was treated with berberine hydrochloride, palmatine hydrochloride, their combination (0.5 mg/mL or 0.5 mg + 0.5 mg/mL) or clotrimazole (0.4 mg/mL) for 6,18, or 30 h. Each group in each time point contained six samples. RNA extraction [24] was performed using Trizol method, concentration purity was determined; reverse transcription was performed using kits from Promega, (Madison, WI, US). *M. canis* cells (5 × 10^9^) were lysed in 1 ml of Trizol Plus reagent and total RNA was isolated according to the manufacturer’s protocol. Total RNA concentration was quantified by determining the optical density at 260 nm. Reverse transcription was performed by mixing 1 μg of RNA with 0.5 μg of oligo (dT)15 Primer and 4 μl of MgCl_2_ (25 mM), 2 μl of reverse transcription 10× Buffer, 2 μl of dNTP Mixture (10 mM), 0.5 μl of Recombinant RNasin Ribonuclease Inhibitor in a sterile tube. Nuclease-free water was added to abtain a final volume of 20 μl. The reaction was incubate at 42 °C for 15 min. Afterward, the samples were heated at 95 °C for 5 min, and incubated at 0 °C for 5 min. The samples were stored at −20 °C until further use.

Real-time PCR was conducted to determine the inhibitory effect of berberine, palmatine, or combined treatment on the differential expression of *M. canis* porphyrin galactose 4 (PGAL4), family of serine hydrolases1 (FSH1), PQ loop repeat protein (PQ-LRP), NADH dehydrogenase subunit 1 (NADH1), ribonucleoprotein (RNP), NADPH-dependent D-xylose reductase (NDR), symbiotic chitinase (SC), and zinc transporter zupT (ZTZ). The primers of related genes were designed according to the gene sequence of *M. canis* listed in Gene Bank (Table 1). 18S ribosomal gene was used as the control. Relative quantification between samples was achieved by the 2 − ⊿⊿CT method [25]. Each reaction was analyzed at least trice.

**Table 1:** Sequences of primer for quantitative RT-PCR of several genes

Gene symbol	Primer	Primer sequence, 5’ → 3’
PGAL4	F	ATGCGCATTGTCCTCAACAG
	R	GGGTGGCCCCAACCA
FSH1	F	TGCTGAGAAGAGACAGGCAAAC
	R	GCTGTCATATTTCTACCGACAACAA
PQ-LRP	F	CCCCCCAGATCATTGAAAACT
	R	CAGACGACGAGGAATTCTAATGATAG
NADH1	F	CCTGCTTTACTTATAGTAGCTTTTGTTACAA
	R	AAATGCTTGGAGTAAACCATAATAACC
RNP	F	GTCCAGGAACTCTTCTCCAAGCACG
	R	GCCACCAAGGTCAGCACCGTATT
NDR	F	TCGAGTTCTGATCGCATGGC
	R	TCGCGGACTGAGAGAGTTCA
SC	F	CTACAGTGGGATACGACCGAGC
	R	TTCCCACCGCGACTGCA
ZTZ	F	CTACCTTACTCGGGCTGGTTACT
	R	TGGTGTGCTGCTATGCTGAT
18S	F	TGGTGCATGGCCGTTCTTA
	R	GGTCTCGTTCGTTATCGCAATT

### Differential expression of M. canis NADH enzyme in the presence of medicine

Approximately 1 mL of *M. canis* cells (5 × 10^9^ CFU/ml) were treated with berberine hydrochloride, palmatine hydrochloride, their combination (0.5 mg/mL or 0.5 mg + 0.5 mg/mL) or clotrimazole (0.4 mg/mL) for 6,18, and 30 h. Shaken culture was washed with PBS twice and then NADH enzyme levels were detected using a Enzychrom kit (Bioassay Systems, CA, USA) .

### Experimental animals

A total of 50 white, male New Zealand rabbits, aged 31 d and weighed 400 g to 450 g, were purchased from the Experimental Animal Centre at Zhejiang University in China. The Bioethics Committee of the Zhejiang Academy of Agricultural Science approved this experiment, and the experimental procedures strictly complied with accepted international rules and regulations. The rabbits were divided into five groups with 10 rabbits in each group by using a simple random method. The two additional groups that were not treated with monomers were classified as the positive control group, which was treated with clotrimazole, and D group, which was treated with DMSO.

### *In-vivo* antifungal assay

At 2 h before the experiment, all of the rabbits received 0.5 mL of cyclophosphamide (20130903) in the middle of the back. Dermatophytosis was induced in the rabbits as previously described [26]. In brief, a 6 cm × 4 cm area of skin was clipped in the middle of the backs of the test rabbits and 1 % of tetracaine hydrochloride (136-47-0) was sprayed on the clipped area. Afterward 1 mL *M. canis* suspension (1.0 × 10^6^ cells) was applied on the marked area by using a sterile pipette tip, and the area was rubbed thoroughly using sandpaper for 10 s. After 3 d, the following treatments were administered to different groups: Group 1(P group) received 1 mL of 1 mg/mL palmatine hydrochloride; Group 2(B group) received 1 mL of 1 mg/mL berberine hydrochloride; Group 3(B-P group) received 1 mg of palmatine hydrochloride and 1 mg of berberine hydrochloride. At 3 d, 1 mL of clotrimazole (0.4 mg/mL) or DMSO was applied topically to C group and D group, respectively. The treatments were repeated each day for 3 d (Table 2). The lesions were evaluated continuously from 1 day post-infection to 17 d post-infection. Clinical evaluation was performed as previously described [27]. In this evaluation, the infected area of skin from each rabbit was divided into four equal quadrants and each area was scored as follows: 0, normal; 1, slightly erythematous patches; 2, well-defined redness, swelling, with bristling hairs, bald patches, or scaly areas; 3, large areas of marked redness, scaling, exposed patches, or ulceration in places; 4, partial damage to the covering and loss of hair; and 5, extensive damage to the covering and complete loss of hair. The scores from the treatment groups were compared. The images of each group were taken on Day 17.

**Table 2:** Experimental schedule of various treatments for each group

Group	No. of Animals	Challenge (day)	Treatment		
			Treatment	Day	Quantity, Volume
P group 1	10	1	Palmatine hydrochloride	3,4,5	1 mg/mL, 1 mL
B group 2	10	1	Berberine hydrochloride	3,4,5	1 mg/mL, 1 mL
B-P group 3	10	1	B-P hydrochloride	3,4,5	2 + 2 mg/mL, 0.5 mL
C group 4	10	1	Clotrimazole	3,4,5	0.4 mg/mL, 1 mL
D group5	10	1	DMSO	3,4,5	1 mL

### Skin histology PAS staining

On the last day of experiment, each group randomly selected 3 samples, sterile scissor cutting skin tissue, after fixation with formalin were embedded in paraffin, and then were observed between groups of inflammation and healing [28] staining PAS.

### Statistical Analysis

The means of real-time PCR, NADH enzyme assay, and *in vivo* antifungal assessment results were compared by using one-way (ANOVA) and Tukey’s HSD test. *P* value < 0.05 was considered statistically significant. Data were expressed as mean ± S.D.

## Results

### *MICs of* berberine or palmatine *and growth curves*

**Fig. 1:**
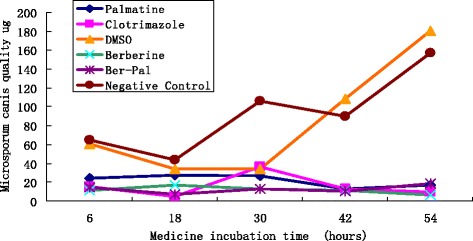
Effects of berberine hydrochloride and palmatine hydrochloride on the growth of *M. canis. M. canis* cells were treated for 54 h with berberine hydrochloride and palmatine hydrochloride or their combination at concentrations of 1 mg/mL, 1 mg/mL, 1 mg/mL + 1 mg/mL respectively; clotrimazole at a concentration of 0.4 mg/mL; or DMSO or Negative control

The MICs were determined after 72 h of growth. The MICs of berberine hydrochloride, palmatine hydrochloride and clotrimazole were 1, 1 and 0.015 mg/mL, respectively. The growth curve of *M. canis* demonstrated that the groups were not significantly different before 18 h but slight difference were observed between treatment groups and DMSO or negative control groups at 6 and 18 h. Growth inhibition caused by berberine hydrochloride, palmatine hydrochloride, or clotrimazole was clearly observed after 42 and 54 h. A slight influence was observed in the growth curve of the DMSO group at 30 h. (Fig. 1)

### Ultrastructure analysis by TEM

**Fig. 2:**
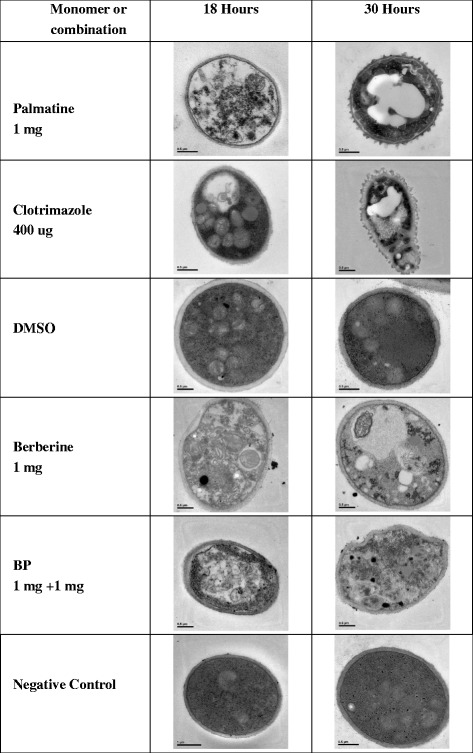
Electron microscopic observation on the morphology of *M. canis* under different medicines or cultivation hours; 1 mg of palmatine or berberine or B-P combination (1 mg + 1 mg) or 400 ug of clotrimazole, DMSO or negative control. The shapes of *M. canis* in different medicine groups with 18 h were abnormal except DMSO or Negative control group. After 30 h incubation, the degree of damage of *M. canis* in medicine groups became more serious than 18 h and still there were no changes in DMSO or Negative control group

In TEM images (Fig. 2), the cell membrane and the cell wall were intact in normal *M. canis* at 18 and 30 h. By contrast, the cell membrane was disrupted in *M. canis* cells treated with berberine hydrochloride, palmatine hydrochloride, combined berberine hydrochloride and palmatine hydrochloride, or clotrimazole at different time points (Fig. 2). DMSO did not evidently influence the growth of *M. canis* after 18 or 30 h.

### Effect of monomers on the expression of related genes

Different inhibitory effects of berberine hydrochloride, palmatine hydrochloride, or combined treatment on energy metabolism and virulence genes of *M. canis* are shown in Figs. 3, 4, 5, 6, 7, 8, 9, 10 and 11, including PGAL4, FSH, PQ-LRP, NADH, RNP, NDR, SC, and ZTZ.

**Fig. 3:**
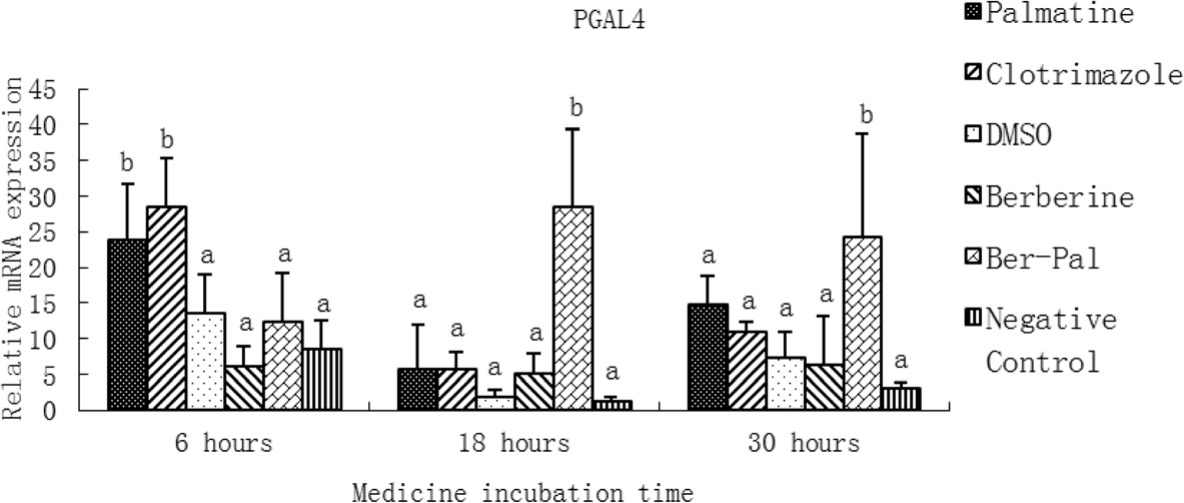
Inhibitory effects of different chemicals on *M. canis* porphyrin galactose 4 *(*PGAL4) gene expression. Different letters in the bars show significantly difference (*P* < 0.05). After 6 h of incubation, the relative mRNA expressions of PGAL4 in the palmatine group and the clotrimazole group were significantly higher than those in other groups (*P* <0.05). After 18 and 30 h, the relative mRNA expression of PGAL4 in the B-P group was significantly higher than that in other groups (*P* < 0.05; Fig. 3)

**Fig. 4:**
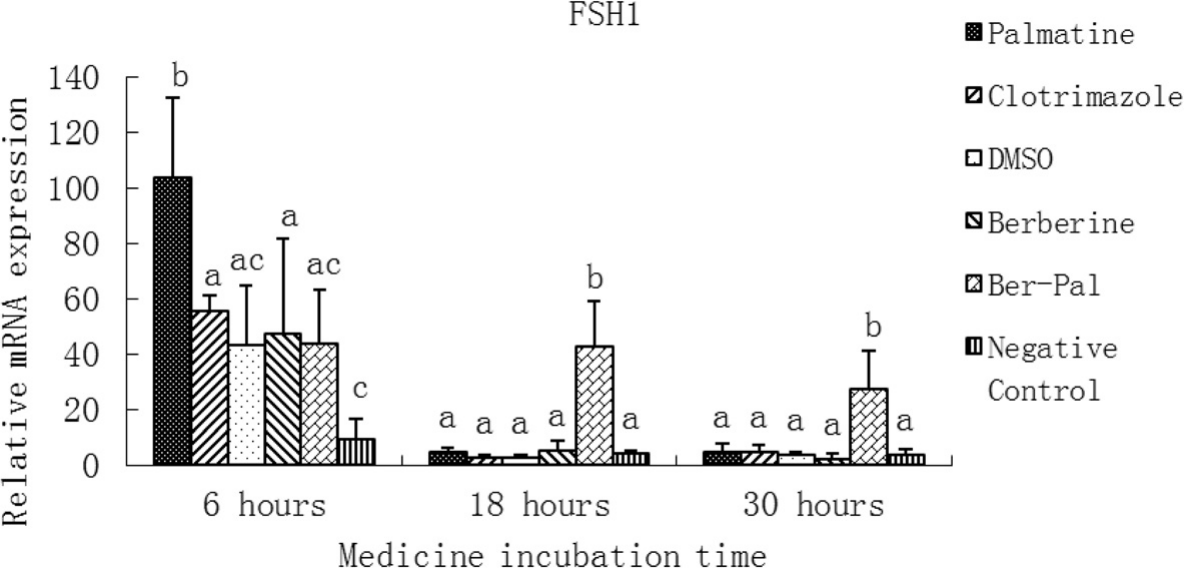
Inhibitory effects of different chemicals on *M. canis* family of serine hydrolases1 (FSH1) gene expression. Different letters in the bars show significantly difference (*P* <0.05). After 6 h of incubation, relative mRNA expression of FSH1 in the palmatine group was significantly higher than that of the other groups (*P* < 0.05). After 18 and 30 h, the highest relative mRNA expression of FSH1 was found in the B-P group (*P* < 0.05)

**Fig. 5:**
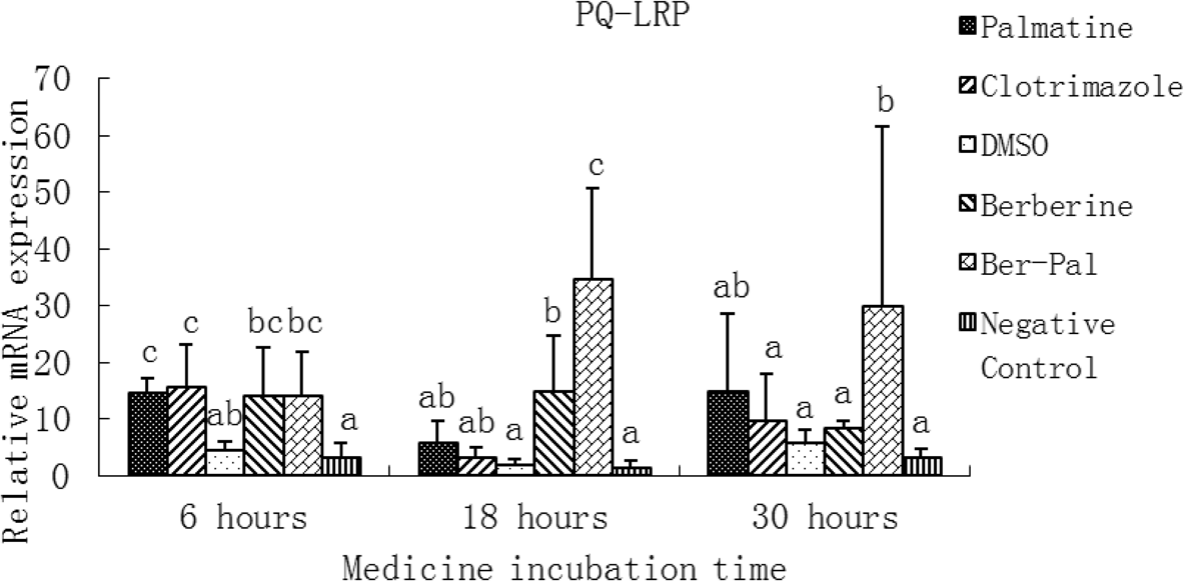
Inhibitory effects of different chemicals on *M. canis* PQ loop repeat protein (PQ-LRP) gene expression. Different letters in the bars show significantly difference (*P* <0.05)After 6 h of incubation, the relative mRNA expressions of PQ-LRP in drug-treated groups were significantly higher than those of DMSO and Negative control groups (*P* < 0.05). After 18 h, the relative mRNA expressions of PQ-LRP in B-P and B groups were significantly higher than those in DMSO and Negative control groups (*P* < 0.05). The relative mRNA expression of PQ-LRP in the B-P group was also significantly higher than that in the berberine group and the clotrimazole group (*P* < 0.05). After 30 h, the relative mRNA expression of PQ-LRP in the B-P group was significantly higher than that in the other groups except in the palmatine group (*P* < 0.05)

**Fig. 6:**
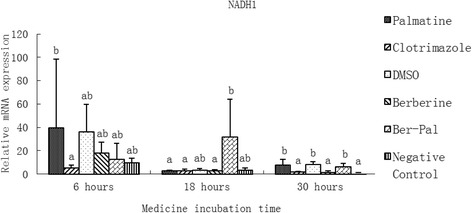
Inhibitory effects of different chemicals on *M. canis* NADH dehydrogenase subunit 1 (NADH 1) gene expression. Different letters in the bars show significantly difference (*P* <0.05)After 6 h of incubation, the relative mRNA expression of NADH1 in the palmatine group was significantly higher than that in the clotrimazole group (*P* < 0.05). After 18 h, the relative mRNA expression of NADH1 in the B-P group was significantly higher than that in the other groups except DMSO and Negative control groups (*P* < 0.05). After 30 h, the relative mRNA expressions of NADH1 in palmatine, DMSO and B-P groups were significantly higher than those in the other groups (*P* < 0.05)

**Fig. 7:**
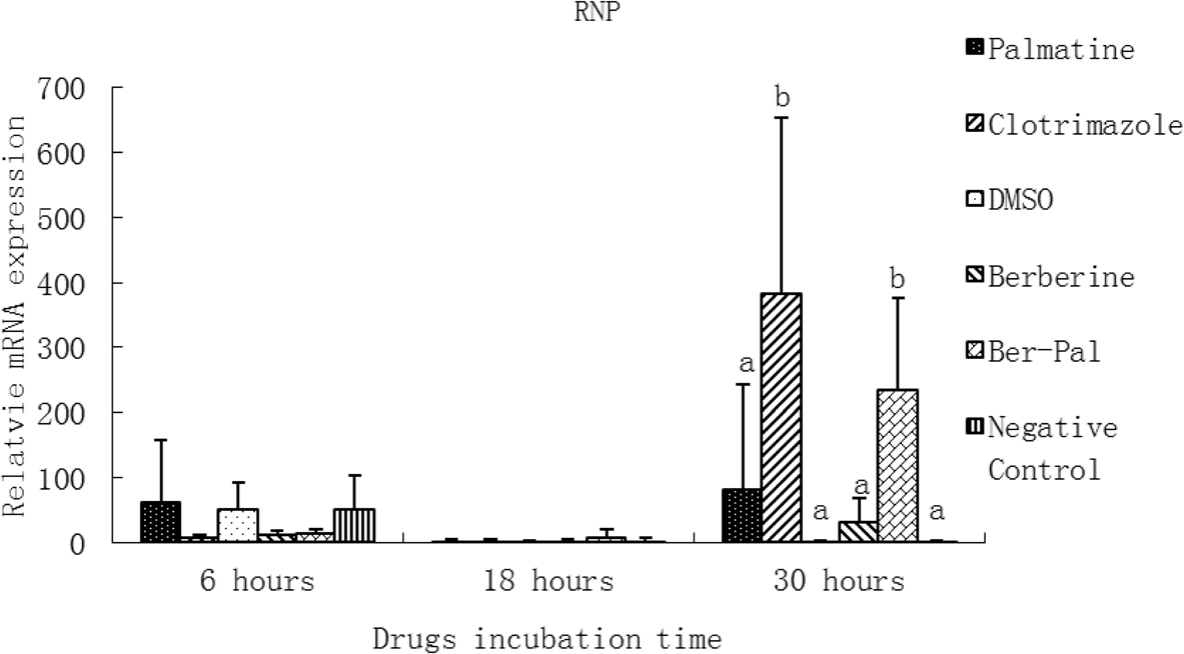
Inhibitory effects of different chemicals on *M. canis* Ribonucleoprotein (RNP) gene expression. Different letters in the bars show significantly difference (*P* <0.05).After 30 h of incubation, the relative mRNA expressions of RNP in B-P and clotrimazole groups were significantly higher than those in the other groups (*P* < 0.05)

**Fig. 8:**
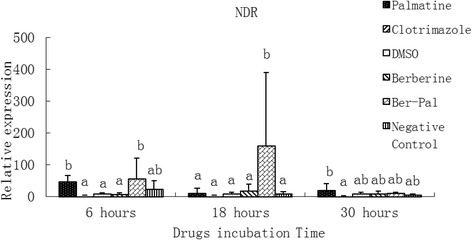
Inhibitory effects of different chemicals on *M. canis* NADPH-dependent D-xylose reductase (NDR) gene expression. Different letters in the bars show significantly difference (*P* <0.05).After 6 h of incubation, the relative mRNA expressions of NDR in palmatine and B-P groups were significantly higher than those in the other groups except the Negative control group (*P* < 0.05). After 18 h, the relative mRNA expression of NDR in the B-P group was significantly higher than that in the other groups (*P* < 0.05)

**Fig. 9:**
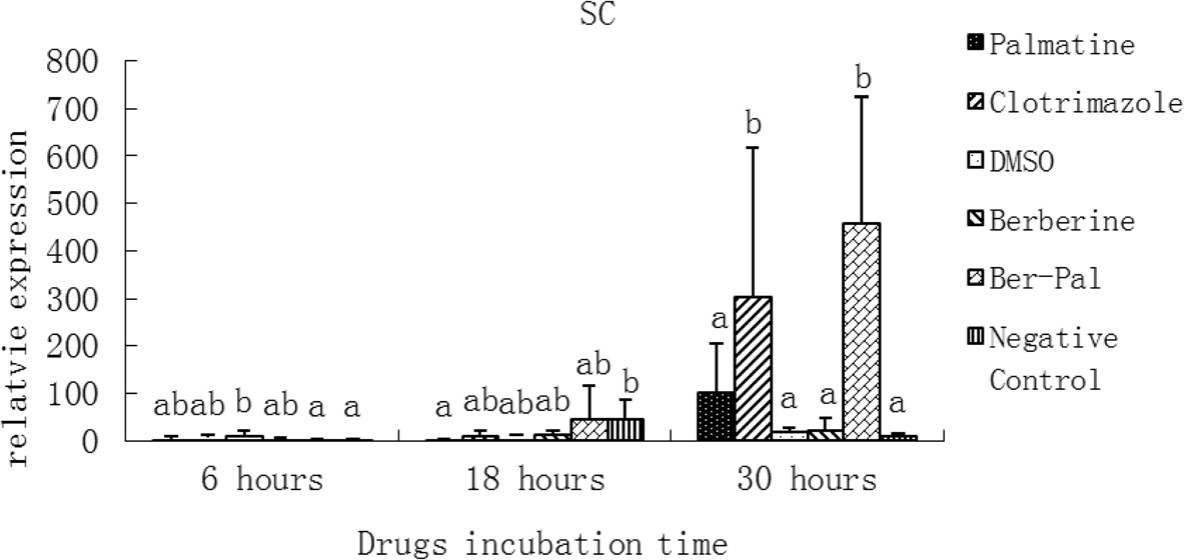
Inhibitory effects of different chemicals on *M. canis* symbiotic chitinase (SC) gene expression. Different letters in the bars show significantly difference (*P* <0.05). After 30 h of incubation, the relative mRNA expressions of SC in B-P and clotrimazole groups were significantly higher than those in the other groups (*P* < 0.05)

**Fig. 10:**
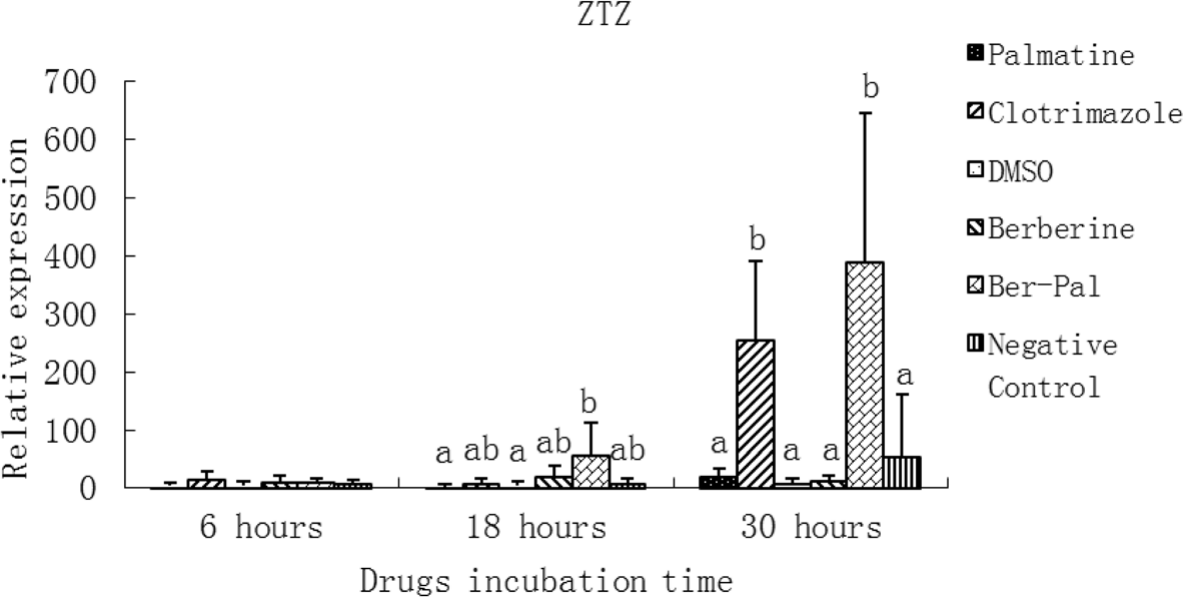
Inhibitory effects of different chemicals on *M. canis* Zinc transporter zupT (ZTZ) gene expression. Different letters in the bars show significantly difference (*P* <0.05).After 30 h of incubation, the relative mRNA expressions of ZTZ in B-P and clotrimazole groups were significantly higher than those in the other groups (*P* < 0.05)

**Fig. 11:**
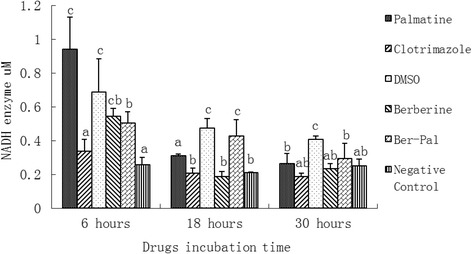
Levels of *M. canis* NADH enzyme in the presence of different drugs. Different letters in the bars show significant difference (*P* < 0.05). After 6 h of incubation, NADH expressions in palmatine, DMSO, berberine and B-P groups were significantly higher than those in clotrimazole and negative groups (*P* <0.05). After 18 h, NADH expressions in DMSO and B-P groups were significantly higher than those in the other groups (*P* < 0.05). NADH expressions in palmatine group was significantly higher than that in clotrimazole, berberine and negative groups (*P* <0.05). After 30 h, NADH expression in the DMSO group was significant higher than that in all the other groups (P < 0.05)

After 6 h of incubation, the relative mRNA expressions of PGAL4 in the palmatime group and the clotrimazole group were significantly higher than those in other groups (*P* < 0.05). After 18 and 30 h, the relative mRNA expression of PGAL4 in the B-P group was significantly higher than that in other groups (*P* < 0.05; Fig. 3).

After 6 h of incubation, relative mRNA expression of FSH1 in the palmatime group was significantly higher than that of the other groups (*P* < 0.05). After 18 and 30 h, the highest relative mRNA expression of FSH1 was found in the B-P group (*P* < 0.05; Fig. 4).

After 6 h of incubation, the relative mRNA expressions of PQ-LRP in drug-treated groups were significantly higher than those of DMSO and negative control groups (*P* < 0.05). After 18 h, the relative mRNA expressions of PQ-LRP in B-P and B groups were significantly higher than those in DMSO and negative control groups (*P* < 0.05). The relative mRNA expression of PQ-LRP in the B-P group was also significantly higher than that in the berberine group and the clotrimazole group (*P* < 0.05). After 30 h, the relative mRNA expression of PQ-LRP in the B-P group was significantly higher than that in the other groups except in the palmatine group (Fig. 5).

After 6 h of incubation, the relative mRNA expression of NADH1 in the palmatine group was significantly higher than that in the clotrimazole group (*P* < 0.05). After 18 h, the relative mRNA expression of NADH1 in the B-P group was significantly higher than that in the other groups except DMSO and negative control groups (*P* < 0.05). After 30 h, the relative mRNA expressions of NADH1 in palmatine, DMSO and B-P groups were significantly higher than those in the other groups (*P* < 0.05; Fig. 6).

After 30 h of incubation, the relative mRNA expressions of RNP in B-P and clotrimazole groups were significantly higher than those in the other groups (*P* < 0.05; Fig. 7).

After 6 h of incubation, the relative mRNA expressions of NDR in palmatine and B-P groups were significantly higher than those in the other groups except the negative control group (*P* < 0.05). After 18 h, the relative mRNA expression of NDR in the B-P group was significantly higher than that in the other groups (*P* < 0.05; Fig. 8).

After 30 h of incubation, the relative mRNA expressions of SC (Fig. 9) and ZTZ (Fig. 10) in B-P and clotrimazole groups were significantly higher than those in the other groups (*P* < 0.05).

### Expression of M. canis NADH enzyme in the presence of different drug treatment

The expression of NADH enzyme from *M. canis* cell treated with different drugs is shown in Fig. 11. After 6 h of incubation, NADH expressions in palmatine, DMSO, berberine and B-P groups were significantly higher than those in clotrimazole and negative groups (*P* <0.05). After 18 h, NADH expressions in DMSO and B-P groups were significantly higher than those in the other groups (*P* < 0.05). NADH expressions in palmatine group was significantly higher than that in clotrimazole, berberine and negative groups (*P* <0.05). After 30 h, NADH expression in the DMSO group was significant higher than that in all the other groups (*P* <0.05).

### *In vivo* antifungal assay

**Fig. 12:**
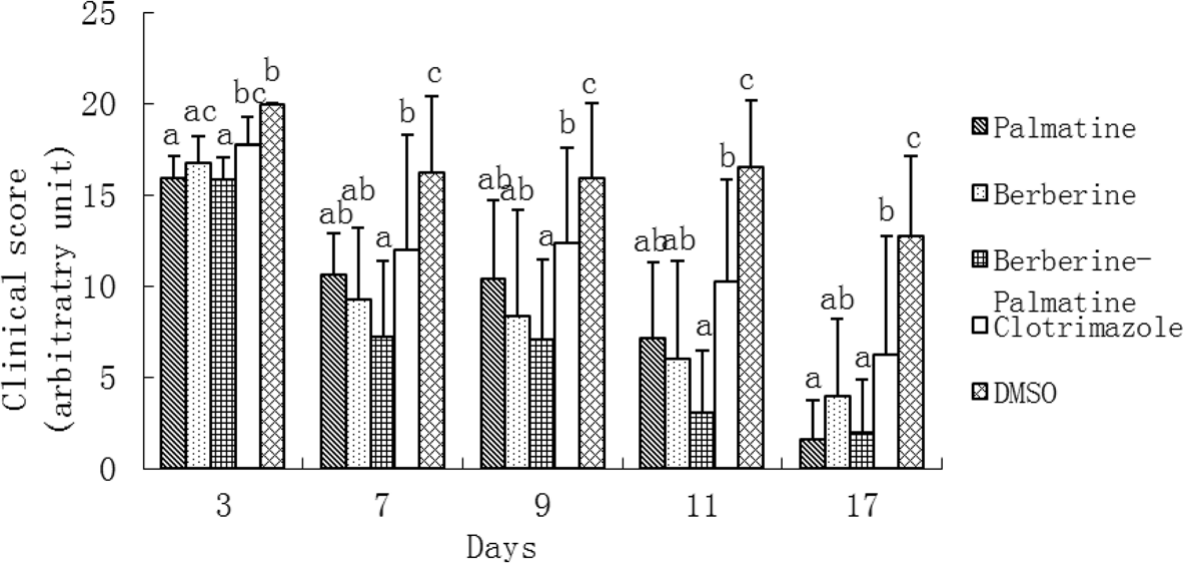
Effects of palmatine hydrochloride (1 mg), berberine hydrochloride (1 mg), palmatine hydrochloride-berberine hydrochloride combination (1 mg + 1 mg), clotrimazole (0.4 mg), and DMSO on induced dermatophytosis in rabbits. Significant differences are indicated at *P* < 0.05. At 3 d, significant recovery was observed in palmatine, berberine, and B-P group (*P* < 0.05). At 7 d, significant recovery of skin lesions was observed in all treatment groups compared with the DMSO group (*P* < 0.05). At 9, 11, and 17 d, significant differences were recorded in B-P and clotrimazole groups compared with the other groups (*P* < 0.05)

**Fig. 13:**
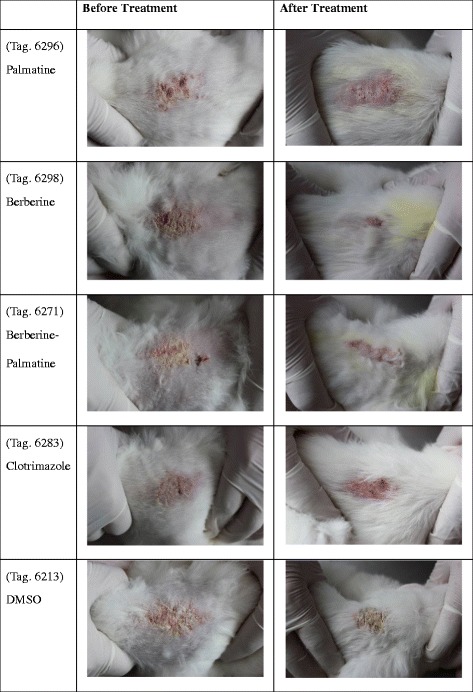
Efficacy of palmatine, berberine, palmatime-berberine or clotrimazole or DMSO against *M.canis* in rabbits. The images were taken on Day 17. Significant differences were recorded in B-P and clotrimazole groups compared with the other groups at Day 17

At 3 d, significant recovery was observed in palmatine, berberine, and B-P group (*P* < 0.05). At 7 d, significant recovery of skin lesions was observed in all treatment groups compared with the DMSO group (*P* < 0.05). At 9, 11, and 17 d, significant differences were recorded in B-P and clotrimazole groups compared with the other groups (*P* < 0.05; Fig. 12, Fig. 13). Although no significant differences were observed among P, B, and B-P groups, the clinical score of B-P group was lower than that of the single monomer groups at 7, 9, and 11 d. No side effects were observed in the drug-treated groups or the DMSO group. No signs of toxicity or local or systemic side effects were also observed in rabbits treated with the monomers. All of the animals were euthanized using standard procedures at the end of the study.

### Skin histology PAS staining results

**Fig. 14:**
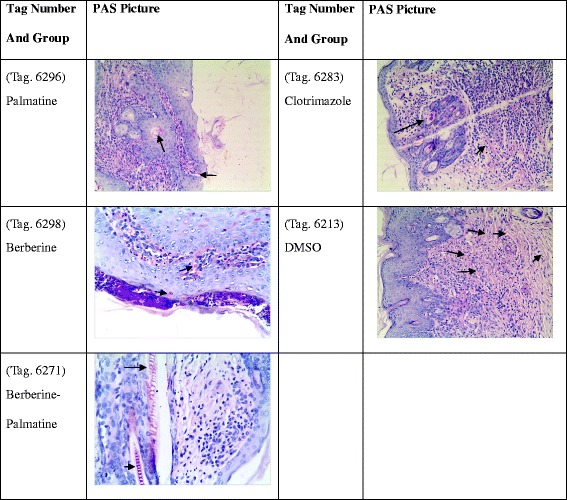
Photos of skin histology PAS staining. The red spot stands for the *M.canis* in the skin. From the figure, it showed that fewer number of *M.canis* occurred in B-P group compared with other groups

Observation under microscope showed that the nucleus of skin cells was blue, red spots standing for the fungus mainly distributed in the surface and cuticle (Fig. 14). The B-P group showed only a small amount of distribution of fungi as well as the clotrimazole group showed few distributions of fungi but DMSO group had a large number of fungi.

## Discussion

Owing to zoonotic transmission of *M. canis*, efforts for effective treatment are necessary. Various parts of traditional medicinal plants were reportedly used against dermatophytosis. *Phellodendron*, a deciduous tree species widely grown in China, is a medicinal plant commonly used to treat various ailments, including gastroenteritis, abdominal pain, diarrhea, abscess, and other inflammations or swellings [29]. Its antibacterial activity against *Staphylococcus aureus*, *S. albus*, α-streptococcus, β-streptococcus, *Proteus* and *Bacillus Dysenteriae*, as well as antifungal activity on *Cryptococcus neoformans* and *Trichophyton purpureatum*, have also been reported [30]. Although reports on the effecacy of medicinal plants against fungi have been presented, studies on their active mechanism are very few. Wang [31] investigated the antifungal mechanisms of cinnamon oil and pogostemon oil complexes on intestinal *Candida* infections. However, data are limited to microscopic evaluation using a scanning electron microscopy (SEM) and TEM.

Berberine hydrochloride and palmatine hydrochloride are the main bioactive components of *Cortex phellodendri*, with quantities of around 0.6 % and 0.3 %, respectively [18]. Berberine and palmatine, are procured from roots of *B. aristata, B. petiolaris, B vulgaris, B.aquifolium, B. thumbergii, B. asiatica* and among Chinese herbs it’s primary sources are *B.sargentiana, Phellodendron amurense* and *Coptis chinensis* from rhizomes and bark respectively. Both are members of a group of alkaloids that have been reported to display various biological and pharmacological activities. Some experiments showed that berberine significantly attenuated *C. pneumoniae* infection-induced VSMC migration (*P* <0.05) and berberine suppressed the protein expressions of MMP3 and MMP9 caused by *C. pneumoniae* infection in a dose-dependent manner (*P* <0.05). *C. pneumoniae* infection-induced increase in the phosphorylation level of Akt at Ser473 was inhibited by the treatment with berberine (*P* <0.05). Taken together, those data suggestted that berberine inhibits *C. pneumoniae* infection-induced VSMC migration by downregulating the expressions of MMP3 and MMP9 via PI3K [32]. Park [33] reported that berberine can inhibit *Candida krusei* with MIC < 1 μg/ml and palmatine can inhibited *Candida tropicalis* with MIC of 16 μg/ml. Zeng [34] found the MIC of berberine to be 640 μg/ml after 16 h incubation based on colony counting of *S. aureus* 2 × 10^7^ CFU/ml. Volleková [20] tested berberine, palmatine and jatrorrhizine for their inhibitory activity against a variety of dermatophytes and two *Candida* species and found that berberine and palmatine exhibit certain antifungal activity (MIC 500 to ≥ 1000 μg/mL). In our study, the MIC of berberine and palmatine was 1 mg/mL and the colony count of *M. canis* was 1.0 × 10^6^ CFU/mL less than 2 × 10^7^ CFU/ml reported by Zeng [34], the colony number and microbes kinds were different might lead to a different MIC. In this study, MIC of clotrimazole was determined to be 15 μg/ml, which is higher than previously reported (0.06 ~ 0.125 μg/ml) [35] and much closer with the results 0.5 ~ 8 μg/ml [36]. We found that the MIC of clotrimazole was lower than the tested monomers. Thus, clotrimazole, which is a very effective antifungal drug, should have higher fungicidal activity. Interestingly, the present study showed that the initial effect of clotrimazole was lower than that of the monomers or their combination (Fig. 12). This result may be caused by the immunosuppressant cyclophosphamide used in the experiments [37], indicating that chemical agents can limit the bioactivity of clotrimazole; as a result, lower efficacy is obtained. Compared with synthetic antifungal medications, natural drugs could evade this problem and may be used safely and effectively.

Electron microscopy showed that certain concentration of monomers could have damaging effects on cell membrane, nucleus and organelles of *M. canis* cells after 18 and 30 h of incubation; thus, the normal growth of the fungi was inhibited. Antifungal mechanisms of monomers possibly rely on this specific effect on fungal cell wall and cell membrane.

Other antifungal mechanisms of drugs were mainly observed on the basis of the following aspects: (1) genetic material quantitative analysis using a laser scanning confocal microscope and scanning image cells [38]; (2) analysis of cell nucleic acid and protein drug effects on fungal DNA synthesis cycle [39]; (3) cell energy metabolism and other effects [40] .

P-GAL4 gene belongs to the GAL4 family, containing only one GAL4 domain, which is specific to fungal species. The protein encoded by GAL4 has different functions in different microorganisms. Hon [41] reported that it can accelerate the synthesis of heme in *Saccharomyces cerevisiae*. Masloff [42] reported that it could regulate fungal reproduction. GAL4 family proteins are also involved in nitrogen metabolism, particularly under medical stress; *M. canis* could increase its metabolism activity to reduce damage and overcome the influence of antifungal drugs. After 6 h of incubation with palmatine and clotrimazole, P-GAL4 expression was up-regulated significantly compared with that in other groups (*P* <0.05). After 18 and 30 h, the expression of P-GAL4 gene in B-P treatment group was also up-regulated significantly compared with other groups (*P* <0.05) (Fig. 3). These results suggested that the antifungal activity of the B-P combination may occur slowly but is more effective than clotrimazole alone. Palmatine possibly up-regulate P-GAL4 activity earlier than berberine. This result is similar to that of Zeng *et al.* [34], in which the antimicrobial activity of berberine was observed after 16 h of incubation.

The protein encoded by FSH1 is a serine hydrolase containing a Ser ⁄ His ⁄ Asp active site. This protein is an esterase that can hydrolyse phosphate ester compounded by analysing its active site and other protein structures [43]. It belongs to a large, multifunctional ab-hydrolase subfamily, the FSH family [44]. As a proteinase, FSH can degrade proteins to amino acids and oligopeptides; as the amidase of an endogenous signal factor, FSH can regulate metabolism. We found that palmatine could up-regulate the expression of FSH1 in as early as 6 h of incubation. After 18 and 30 h, the antifungal activity of B-P combination was evident, and FSH1 expression was significantly upregulated (Fig. 4). The function of FSH1 could be the same as that of P-GAL4.

The PQ-LRP protein is a membrane-binding protein containing a pair of ring structures. As a secondary signal, the second ring is quite important to cysteine vector localization in the lysosome [45] and it is useful for the anabolic metabolism of cysteine. Fedorova [46] identified the PQ-LRP gene when they analyzed the genomic DNA of *Aspergillus fumigatus*; they proposed that the PQ-LRP protein could be a general component of fungal cells and thus, related to fungal growth and development. PQ-LRP gene up-regulation could enhance cysteine metabolism; PQ-LRP protein could produce cysteine by decomposing disulfide bond of proteins, which increase the pathogenicity of *M. canis*. After 6 h of incubation, PQ-LRP expressions were up-regulated in all of the drug-treated groups compared with NC control. After 18 h, PQ-LRP expressions in B and B-P groups were up-regulated compared with those in the NC group. After 30 h, PQ-LRP expressions in B-P group was significant higher than that in other groups except the P group (Fig. 5).

NADH1, which is encoded in the mitochondria, is an important part of the respiratory chain. NADH1 passes electrons in the respiratory chain of mitochondrial oxidation to harness energy through ATP. B-P combination enhanced the expression of NADH1 mainly after 18 h of incubation (Fig. 6). The results of NADH at the enzyme level were slightly different from that obtained from gene expression. However, after 30 h, the results were almost same. P, B-P combination, and NC were significantly higher than the other groups (*P* <0.05) based on real-time PCR. This result was also observed at the enzyme level (Fig. 11).

Ribonucleoproteins (RNPs) are RNA-associated proteins that combine with mRNA and non-coding RNAs to form RNP complexes. In the context of structured RNPs, the activity and stability of many RNAs are regulated post-transcriptionally. The composition of an RNP dictates RNA fate, reflecting aberrations that subject the RNA to quality-control degradation pathways. After 6 h to 18 h of incubation, no significant difference in terms of RNP expression was found between the groups, which means that the influence of drugs on RNP expression were minute. After 30 h of incubation with the clotrimazole and combined group, the mRNA expression of RNP was significantly higher than that of the other groups (*P* <0.05) (Fig. 7).

Xylose reductase is an intracellular enzyme commonly found in yeast and filamentous fungi. This enzyme occurs in the cytoplasm of microorganisms, where it catalyzes the first step of xylose metabolism by reducing xylose to xylitol with the concomitant oxidation of NAD(P)H to NAD(P) + [47]. After 6 h of incubation, the mRNA expression of NDR in P and B-P group were significantly higher than that of the other groups except for the NC group. After 18 h of incubation, the mRNA expression of NDR in the B-P group was significantly higher than the other groups (*P* <0.05) (Fig. 8), which is similar with the results of NADH1 real time PCR after 18 h.

Chitinases are enzymes that cleave the β-(1,4) glycosidic bond of chitin, a structural component in fungal cell walls. The carbohydrate-active enzyme (CAZy) glycoside hydrolase family 18 (GH18), as defined by amino acid sequence similarity [48], contains a large number of chitinases expressed in prokaryotes and eukaryotes. It is subdivided into two subfamilies, bacterial-type and plant-type family 18 chitinases, according to sequence similarity, active site construction, preferred activity (exo-chitinases versus endochitinases, respectively), and occurrence in different organisms. It has been hypothesized that bacterial-type chitinases in fungi and bacteria are used to process chitin as a carbohydrate [46]. After 30 h of incubation, the mRNA expressions of SC in PC and B-P groups were significantly higher than those in the other groups (*P* <0.05) (Fig. 9).

Zinc transporter zupT (ZTZ) mediates zinc uptake. It transport zinc from within the cell, either out of the cell across the plasma membrane or into intracellular compartments, which reduces cytosolic concentrations [49]. After 30 h of incubation, the mRNA expressions of ZTZ in PC and B-P groups were significantly higher than those in the other groups (*P* <0.05) (Fig. 10).

Real-time PCR results showed that palmatine likely induced the up-regulation of certain gene expressions earlier than berberine. By contrast, berberine possibly regulated antifungal activity, especially after 18 h, in which B-P shortened the life cycle of *M. canis* and eradicated this fungus. Clotrimazole could also increase the mRNA expressions of PQ-LRP, PGAL4 and FSH1 for a short period but failed to maintain such high expression.

*In vivo* study, it was observed that the lesions in the B-P treatment groups started to recover from day 7. No significant differences between P, B and B-P treatment groups were found, while average score of B-P group was lower than P, B group from day 7. Treatment with clotrimazole subsided skin lesions, but there was no significant difference when compared with P and B group but showed significant difference when compared with B-P group from day 7. The results of PAS were also consistent with the results of clinical experiment. Clotrimazole is well-documented antifungal agent, and the performance of B-P in this study was somewhat better than the performance of clotrimazole.

The monomers from *P. amurense* possibly exhibited antifungal activities by disrupting fungal cell wall and cell membrane and by increasing the expressions of energy metabolic genes; thus, the life cycle of *M. canis* was shortened. Our results showed that the mixture of palmatine hydrochloride and berberine hydrochloride could be used effectively to treat rabbit dermatomycosis caused by *M. canis*.

## Conclusion

These results provide a comprehensive view of the mechanism of berberine and palmatine in anti- *M. canis* activity.
